# Association between HOMA-IR and Lung Function in Korean Young Adults based on the Korea National Health and Nutrition Examination Survey

**DOI:** 10.1038/s41598-017-11337-3

**Published:** 2017-09-15

**Authors:** Young Bok Lee, Young Soo Kim, Dong-Hee Lee, Hee Yeon Kim, Jae-Im Lee, Hyo-Suk Ahn, Tae Seo Sohn, Tae-Kyu Lee, Jae Yen Song, Chang Dong Yeo, Mihee Hong, Kyungdo Han, Seong Cheol Jeong, Hiun Suk Chae

**Affiliations:** 1 Epidemiology Study Cluster of Uijeongbu St. Mary’s Hospital, Uijeongbu St. Mary’s Hospital, College of Medicine, The Catholic University of Korea, Seoul, South Korea; 2 Department of Biostatistics, College of Medicine, The Catholic University of Korea, Seoul, Korea

## Abstract

Metabolic syndrome, including obesity and insulin resistance, has been reported to lower lung function in elderly subjects with asthma or chronic obstructive lung disease. This study aimed to find the association between lung function and insulin resistance in Korean young adults. This study used data from the Korean National Health and Nutrition Examination Survey 2011–2013, which is a representative sample of the Korean population. A total of 1,922 young adults aged 19 to 40 were included in the analysis. The association between lung function test and insulin resistance was evaluated. Weighted logistic regression analyses showed a significant negative correlation of insulin resistance with FVC% predicted (correlation coefficient γ = −0.130, *P* < 0.0001), FEV_1_% predicted (γ = −0.074, P = 0.004) and FEV_1_/FVC ratio (γ = −0.059, *P* = 0.019) in young adults, especially in subjects without asthma (γ for FVC% predicted, FEV_1_% predicted and FEV_1_/FVC ratio = −0.138, −0.092, and −0.061, respectively). This study demonstrates an inverse correlation between insulin resistance and lung function in Korean young adults. Young adults with preclinical insulin resistance have a higher risk of impaired lung function.

## Introduction

Insulin resistance is defined as a condition in which higher than normal insulin concentrations are needed to achieve normal metabolic responses or normal insulin concentrations fail to achieve a normal metabolic response^1^. The failure of insulin to stimulate glucose transport into its target cells plays a central etiological role in metabolic syndrome, which includes abdominal obesity, hypertriglyceridemia, low high-density lipoprotein-cholesterol, elevated blood pressure, and hyperglyceridemia^1^.

A negative correlation between lung function and metabolic syndrome has recently been proposed in several studies. In Forno’s study, adolescents with asthma and metabolic syndrome showed significantly decreased pulmonary function compared to adolescents with asthma alone^2^. Yeh *et al*. reported that subjects with metabolic syndrome had lowered lung function^3^. Obesity, a component of metabolic syndrome, was reported as a risk factor for decreased pulmonary function in asthma patients^2,4^. However, the metabolic components playing a key role in lower lung function have not been clearly elucidated.

The correlation between lung function and insulin resistance that is an underlying pathophysiology leading to metabolic syndrome remains unclear. Forno *et al*. reported that insulin resistance is associated with lower lung function in adolescents^2^. Yamamoto *et al*. reported that impaired lung function was associated with higher risk of metabolic syndrome independent of insulin in middle aged Japanese population^5^. In another study, Paek *et al*. reported that Korean patients with chronic obstructive pulmonary disease (COPD) had higher risk of metabolic syndrome; however, glucose level was not associated with low lung function^6^. In another Korean study, Park *et al*. demonstrated that obesity was associated with COPD; however, fasting glucose level was not associated, and insulin resistance was not determined^7^.

The present study aimed to find the association between insulin resistance and lung function in young adults under age 40, in whom COPD is not prevalent. We divided subjects into two groups according to the presence of asthma that might affect lung function test using the data from the 2011–2013 Korean National Health and Nutrition Examination Survey (KNHANES V-2, V-3, and VI-1). This study demonstrates the association between preclinical low lung function and subclinical insulin resistance in a healthy population.

## Results

### Demographics

The characteristics of the study participants are summarized in Table 1. Among 1,922 participants aged 19 to 40 years with lung function test, 216 had a medical history of asthma. No significant differences were observed between the asthma and non-asthma groups regarding mean age, sex, exercise, area of residence, house-hold income, weight, height, BMI, waist circumference, energy intake, serum levels of glucose and insulin, HOMA-IR, and energy intake. However, the percentage of current smokers was significantly lower in the no-asthma group (34.9 ± 1.3%) than in the asthma group (53.7 ± 3.7%, P < 0.0001). The percentage of heavy current alcohol users (>30 g/day) was significantly higher in the asthma group (21.2 ± 3.0%) than in the no-asthma group (13.1 ± 0.9%, P = 0.0029). The prevalence of atopic dermatitis and allergic rhinitis were significantly higher in the asthma group (P < 0.0001). This finding was expected as atopic dermatitis and allergic rhinitis are known to be common in asthma patients.

**Table 1:** Demographics of participants.

	No-asthma group n = 1706	Asthma group n = 216	*P* value
Sex (Male, %)	57.2 ± 1.3	64 ± 3.7	0.0635
Age (Years)	31.2 ± 0.2	30.7 ± 0.5	0.2868
Exercise (Yes, %)	26.8 ± 1.3	22.3 ± 2.9	0.1641
Place (Rural, %)	12.0 ± 2.1	14.6 ± 3.2	0.3827
Current smoker (Yes, %)	34.9 ± 1.3	53.7 ± 3.7	<0.0001
Current alcohol use (>30 g/day, %)	13.1 ± 0.9	21.2 ± 3	0.0029
Income (Less than 25%, %)	7.1 ± 0.8	4 ± 1.5	0.1104
Atopic dermatitis (Yes, %)	4.2 ± 0.5	8.1 ± 2.3	<0.0001
Allergic rhinitis (Yes, %)	30.8 ± 1.6	53.6 ± 4.4	<0.0001
Height (cm)	167.7 ± 0.2	168.7 ± 0.6	0.1
Weight (kg)	68.5 ± 0.3	68.8 ± 1	0.7494
Body mass index (kg/m^2^)	24.2 ± 0.1	24.1 ± 0.3	0.6181
Waist circumference (cm)	82.1 ± 0.3	82.4 ± 0.9	0.7356
FVC (L)	4.2 ± 0.0	4.3 ± 0.1	0.6624
FVC % predicted	95.1 ± 0.3	93.8 ± 0.7	0.0891
FEV_1_ (L)	3.5 ± 0.0	3.5 ± 0.1	0.3009
FEV_1_% predicted	93.8 ± 0.3	90 ± 0.8	<0.0001
FEV_1_/FVC ratio	0.84 ± 0.00	0.8 ± 0	0.0002
Glucose (mg/dL)	91.0 ± 0.2	90 ± 0.6	0.1355
Insulin (μIU/mL)	9.6 (9.4,9.9)	9.5(8.9–10.1)	0.4318
HOMA IR	2.2 (2.1,2.2)	2.1(2–2.2)	0.644
Energy intake (kcal/day)	2039.9 ± 26.8	2123.2 ± 74.9	0.2826
Fat intake (% of calorie)	20.8 ± 0.3	20.5 ± 0.7	0.695

HOMA-IR, homeostasis model of assessment of insulin resistance; FVC, forced vital capacity; FEV1, forced expiratory volume in 1 second.

### Association between lung function and insulin resistance

The results of multivariate analysis of the relationships between insulin resistance and lung function measurements in total population, the no-asthma group, and the asthma group are shown in Table 2. The levels of insulin and insulin resistance (HOMA-IR) negatively correlated with FVC% predicted, FEV1% predicted, and FEV1/FVC ration in total population and the no-asthma group (Table 2). However, the inverse correlation between insulin resistance and lung function was not statistically significant in the asthma group. Each unit increase in serum level of insulin and HOMA-IR value was significantly associated with lower FVC% predicted (r coefficient = −0.130 for insulin level and −0.133 for HOMA-IR, P < 0.0001 for both), FEV_1_% predicted (r coefficient = −0.074 for insulin level and −0.078 for HOMA-IR, P = 0.004 and P = 0.0025, respectively) and FEV_1_/FVC ratio (r coefficient = −0.058 for insulin level and −0.045 for HOMA-IR, P = 0.0019 and P = 0.0744, respectively) in the total population group. The no-asthma group showed stronger negative association between lung function and insulin resistance than total population (r coefficient for FVC% predicted = −0.138 for insulin level and −0.128 for HOMA-IR, P < 0.0001 for both, r coefficient for FEV_1_% predicted = −0.092 for insulin level and −0.084 for HOMA-IR, P = 0.0019 and P = 0.0041, respectively, and r coefficient for FEV_1_/FVC ratio = −0.061 for insulin level −0.081 and for HOMA-IR, P = 0.0269 and P = 0.0026, respectively).

**Table 2:** Correlation analysis between insulin resistance and lung function in participants.

Insulin	Total population		No-asthma group		Asthma group		*P* value for interaction
	r	*P* value	r	*P* value	r	*P* value	
FVC	0.02246 (−0.0227, 0.0675)	0.3622	0.013 (−0.0349, 0.0608)	0.6145	−0.04 (−0.1738, 0.0953)	0.5528	0.4472
FVC% predicted	−0.13069 (−0.1748, −0.0861)	<**0**.**0001**	−0.138 (−0.1846, −0.0907)	**<0.0001**	−0.107 (−0.2383, 0.0282)	0.175	0.7497
FEV_1_	0.00286 (−0.0422, 0.048)	0.9122	−0.007 (−0.0548, 0.0409)	0.8077	−0.028 (−0.1621, 0.1072)	0.6549	0.7504
FEV_1_% predicted	−0.07418 (−0.1189, −0.0292)	**0.004**	−0.092 (−0.1393, −0.0443)	**0.0019**	0 (−0.1348, 0.1348)	0.9981	0.2927
FEV_1_/FVC ratio	−0.05889 (−0.1037, −0.0138)	**0.019**	−0.061 (−0.1096, −0.0132)	**0.0269**	0.031 (−0.1042, 0.1651)	0.6889	0.3393
**HOMA-IR**	**r**	***P*** **value**	**r**	***P*** **value**	**r**	***P*** **value**	***P*** **value for interaction**
FVC	0.00736 (−0.0378, 0.0524)	0.7641	0.032 (−0.0159, 0.0797)	0.2241	−0.053 (−0.1864, 0.0823)	0.4191	0.224
FVC% predicted	−0.13301 (−0.177, −0.0884)	<0.**0001**	−0.128 (−0.1748, −0.0806)	**<0.0001**	−0.162 (−0.2904, −0.0279)	**0.0313**	0.6112
FEV_1_	−0.0085 (−0.0536, 0.0366)	0.7423	0.007 (−0.0409, 0.0548)	0.8074	−0.033 (−0.167, 0.1022)	0.6024	0.5597
FEV_1_% predicted	−0.07843 (−0.1231, −0.0334)	**0.0025**	−0.084 (−0.1313, −0.0363)	**0.0041**	−0.025 (−0.1592, 0.1101)	0.7272	0.5217
FEV_1_/FVC ratio	−0.04549 (−0.0904,−0.0004)	0.0744	−0.081 (−0.1284, −0.0333)	**0.0026**	0.049 (−0.0863, 0.1825)	0.5216	0.1724

r, correlation coefficient; HOMA-IR, homeostasis model of assessment of insulin resistance; FVC, forced vital capacity; FEV1, forced expiratory volume in 1 second.

We next performed multiple logistic regression analyses between HOMA-IR and lung function in comparison with the asthma group and no-asthma group (Table 3). The no-asthma group showed that each unit increase of HOMA-IR was significantly associated with FEV_1_% predicted (β coefficient = −4.467, P < 0.0001) and FVC% predicted (β coefficient = −3.892, P < 0.0001) after adjustment for age, sex, and BMI in Modele 1. After fully adjusting for age, sex, BMI, smoking status, drinking status, and exercise (Model 2), the β coefficient (in the no-asthma group) was −4.267 for FEV_1_% predicted (P < 0.0001), −3.656 for FVC% predicted (P < 0.0001), and −0.007 for FEV_1_/FVC ratio (P = 0.004) in the no-asthma group. However, the inverse correlation between lung function and HOMA-IR was not statistically significant in the asthma group.

**Table 3:** Regression coefficients of dependent variables of lung function.

	Lung function	HOMA-IR						*P* value for interaction
		No- asthma group			Asthma group			
		Beta	SE	*P* value	Beta	SE	*P* value	
Model 1	FEV_1_% predicted	−4.467	0.793	**<0.0001**	−1.399	2.164	0.5185	0.6113
	FVC% predicted	−3.892	0.719	**<0.0001**	−3.917	2.315	0.0916	0.6655
	FEV_1_/FVC ratio	−0.007	0.004	0.0985	0.02	0.014	0.1403	0.1962
Model 2	FEV_1_% predicted	−4.267	0.811	**<0.0001**	−1.221	2.159	0.5721	0.5658
	FVC% predicted	−3.656	0.736	**<0.0001**	−3.368	2.414	0.1638	0.6567
	FEV_1_/FVC ratio	−0.007	0.004	0.0864	0.018	0.012	0.1592	0.1478

Data are presented as β coefficients and SE (standard error). Model 1: adjusted for age, sex, and BMI; Model 2: adjusted for age, sex, BMI, smoking status (current smoker), drinking status (>30 mg/day), and exercise status.

HOMA-IR, homeostasis model of assessment of insulin resistance; FVC, forced vital capacity; FEV1, forced expiratory volume in 1 second.

### Linear correlation between HOMA-IR and lung function in the no-asthma group

We next stratified participants into four quartiles according to HOMA-IR level in both no-asthma and asthma groups (Table 4). In the no-asthma group, the mean value of FEV_1_% predicted was 96.2026% for HOMA-IR quartile 1, 94.8399% for HOMA-IR quartile 2, 92.7097% for HOMA-IR quartile 3, and 91.8195% for HOMA-IR quartile 4. The negative linear correlation was statistically significant between HOMA-IR and FEV_1_% predicted (P < 0.0001). The mean values of FVC% predicted according to HOMA-IR quartiles were 97.4197%, 95.8775%, 94.2153%, and 93.1292%, respectively (P < 0.0001) in the no-asthma group. Although the *P* value for interactions between the two groups was not statistically significant, the difference was observed that there was no significant linear correlation between lung function and HOMA-IR in the asthma group (Table 4).

**Table 4:** Mean of FEV1%, FVC% and FEV1/FVC ratio according to quartiles of HOMA-IR.

HOMA-IR	FEV_1_% predicted		FVC% predicted		FEV_1_/FVC ratio	
	No-asthma	Asthma	No-asthma	Asthma	No-asthma	Asthma
Quartile 1	96.2 ± 0.5	88.1 ± 1.5	97.4 ± 0.5	93.9 ± 1.6	0.841 ± 0.003	0.803 ± 0.014
Quartile 2	94.8 ± 0.6	90.6 ± 1.4	95.9 ± 0.6	94.8 ± 1.4	0.841 ± 0.003	0.814 ± 0.010
Quartile 3	92.7 ± 0.6	91.4 ± 1.6	94.2 ± 0.5	93.8 ± 1.4	0.837 ± 0.003	0.828 ± 0.008
Quartile 4	91.8 ± 0.6	88.9 ± 1.6	93.1 ± 0.5	92.3 ± 1.5	0.838 ± 0.003	0.820 ± 0.010
*P* value	<0.**0001**	0.4004	<0.**0001**	0.7446	0.7131	0.4019
*P* value for interaction		0.187		0.8567		0.35

HOMA-IR, homeostasis model of assessment of insulin resistance; FVC, forced vital capacity; FEV1, forced expiratory volume in 1 second.

## Discussion

To the best of our knowledge, this study is the first to demonstrate a negative correlation between insulin resistance and lung function in a representative sample of Korean young adults. This study showed that HOMA-IR is associated with lower FEV_1_% predicted, FVC% predicted and FEV_1_/FVC ratio. In addition, lung function was significantly affected by HOMA-IR level; individuals with higher HOMA-IR (greater insulin resistance) had significantly lower lung function. This study has a unique point in that it revealed an independent association between subclinical insulin resistance and lung function based on a database of young adults.

There have been several studies documenting the association between overt diabetes mellitus and impaired lung function^8–10^. Several studies have evaluated the association between insulin resistance and lung function in adult and elderly participants^11–13^. Kim *et al*. reported that insulin resistance was associated with low FVC in both obese and non-obese middle-aged Koreans over 30 years (mean age >50 years)^13^. Lawlor *et al*. demonstrated that HOMA score was inversely associated with FEV_1_ and FVC in British women between 60 and 79 years of age^11^. Lazarus *et al*. also reported inverse associations between insulin resistance and FEV_1_ and FVC in males aged 21 to 80 in Boston, Massachusetts area^12^. However, age is a very important determining factor in lung function. Decrease in muscle mass with age results in muscle weakness and decreases in FVC and FEV_1_. The high prevalence of COPD in elderly individuals might also have acted as a confounding factor in the previous studies. Thus, the association between lung function and insulin resistance without comorbidities needs to be evaluated in young adults.

Asthma is characterized by airway obstruction like COPD and is common in people under 35 years of age, unlike COPD, which is an elderly disease. In a previous study, an inverse correlation between metabolic syndrome and lung function was observed in asthma patients^4^. However, in this study, the asthma group did not show a significant inverse correlation between lung function and insulin resistance. This discrepancy might be explained by the difference in subjects. Unlike previous studies in which asthma subjects were included by medical chart review, the asthma group in this study was collected by questionnaire survey with self-reported asthma. Although the FEV_1_% predicted and FEV_1_/FVC ratio of the asthma group were lower than those of the no-asthma group, the FVC, FEV_1_, FVC% predicted, FEV_1_% predicted, and FEV_1_/FVC ratio in both groups were within the normal range, without any evidence of impaired lung function. Another possible explanation is that the remaining confounding factors could affect lung function in the asthma group, such as smoking, drinking status, and the presence of allergic diseases. Impaired lung function seemed to have a negative correlation with insulin resistance in the asthma group; however, no statistical significance was found, likely due to the other confounding factors.

Until now, the mechanisms of lower lung function by insulin resistance have not been clearly elucidated. Several possible mechanisms have been reported. Kim *et al*. demonstrated that HOMA-IR was significantly associated with bronchial hyper-reactivity^14^. In an vitro study, Dekkers *et al*. evaluated that insulin-induced hypercontractililty after eight days of tissue culture of bovine smooth muscle cells^15^. Conversely, the influence of lung function on systemic insulin resistance has also been reported. Sarlus *et al*. revealed the brain gene expression toward induction of insulin resistance that was induced by airway-associated inflammation^16^. Thuesen *et al*. reported that insulin resistance was a predictor of asthma-like symptoms in adults^17^. The mechanisms that link insulin resistance with impaired lung function should be elucidated in future studies.

Obesity is a well-known risk factor for impaired lung function and is associated with insulin resistance. Higher BMI might be a confounding factor between insulin resistance and lung function^18^. BMI has a positive correlation with FEV_1_/FVC and a negative correlation with FEV_1_^19^. One possible mechanism to explain decreased lung function caused by obesity is decreased lung volume, especially expiratory reserve volume and functional residual capacity^20^. The ineffectiveness of respiratory muscles in obesity also results in decreased lung function. Obesity is considered a chronic inflammatory state and is associated with insulin resistance, endothelial dysfunction, systemic arterial hypertension, and dyslipidemia^20^. However, Thuesen’s study demonstrated that the effect of insulin resistance was stronger than that of obesity^17^.

Our study has several limitations. First, the definition of no-asthma was based on a self-reported system. Second, the present study did not compare other components of metabolic syndrome such as diabetes mellitus and obesity in association with lung function. The relationship between insulin resistance and lung function could be strengthened by including these variables in future studies.

In conclusion, data from the Korean National Health and Nutrition Examination Survey revealed that insulin resistance is negatively correlated with lung function in young Korean adults. This study is important in understanding the association between insulin resistance and lung function in the young Korean population. Our results suggest the importance of early management and education on metabolic syndrome for preservation of pulmonary function, one of the most important determinants of comorbidity and mortality.

## Materials and Methods

### Study population and data collection

We used data from the Fifth Korean National Health and Nutrition Examination Survey (KNHANES V-2, V-3, and VI-1), which was conducted from January 2011 to December 2013, by the Korea Centers for Disease Control and Prevention. The study design followed the tenets of the Declaration of Helsinki for biomedical research. This survey was conducted after approval from the Institutional Review Board of the Korea Centers for Disease Control and Prevention (IRB No. 2011-02CON-06-C, 2012-01EXP-01-2C, and 2013-07CON-03-4C). It was performed using a rolling sampling design involving a complex, stratified, multistage, probability cluster survey of a representative sample of the civilian population of South Korea. A total of 192 sampling units were randomly selected from primary sampling units encompassing the target population. Each sampling unit contained 20 households, with a total of 3800 households surveyed in one year. The Korean National Health and Nutrition Examination Survey V-2, V-3, and VI-1 were conducted by four survey teams. The survey consisted of three components: a health interview, a nutrition interview, and health examination.

All questionnaires were administered in person either by physicians or by trained interviewers at the participants’ homes. Participants had the right to refuse participation according to the National Health Enhancement Act. All participants who agreed to take part provided written informed consent. The Korea Centers for Disease Control and Prevention obtained agreement from participants to use blood samples collected during the health interview survey for further research.

We excluded participants younger than 19 years or older than 40 years and participants without a pulmonary function test. A total of 1,922 participants were included in this analysis.

### Anthropometry and laboratory measurements

Trained medical staff performed the physical examinations according to standardized procedures. The health interview and health behavior surveys included well-established questions to determine the demographic and socioeconomic characteristic of the subjects. The surveys included questions on age, residence, family income, education level, occupation, marital status, smoking habit, alcohol consumption, exercise, previous and current disease, and family disease history. Smoking status was divided into three categories: current smoker, ex-smoker, or nonsmoker. A heavy alcohol consumer was categorized as one who drinks more than 30 g per day. Exercise status was divided into yes or no (regular exercise ≥ once a week). Residential area was categorized as urban or rural. Body weight was measured to the nearest 0.1 kg with subjects wearing light indoor clothing and height was measured to the nearest 0.1 cm without shoes. Body mass index (BMI) was calculated as the ratio of weight in kilograms to height in square meters. After overnight fasting, blood samples were obtained from participants’ antecubital veins. Levels of glucose and insulin were measured enzymatically using a Hitachi Automatic Analyzer 7600 (Hitachi, Chiyoda-ku, Japan). Daily calorie intake was monitored by 24-h food recall and analyzed using CAN-Pro 3.0 software (Korean Nutrition Society, Seoul, Korea).

### Definition of asthma

Asthma was defined as a self-reported diagnosis by a physician in the health interview surveys. The questionnaire contained questions on physician-diagnosed asthma, wheezing, and use of asthma medication. We used the following survey questions in this study: “Have you ever been diagnosed with asthma by a doctor”?, “Have you experienced wheezing or whistling in your chest at any time in the last 12 months”?, “Are you currently taking any medicine (including inhalers, aerosols or tablets) for asthma”? Participants who answered “Yes” to these questions were considered to have been diagnosed with asthma by a doctor. The questionnaire was validated and is generally used in epidemiologic studies of asthma^21–23^.

### Lung function tests

Lung function was measured using a dry rolling seal spirometer (model 2130, SensorMedics, Yorba Linda, CA) according to the American Thoracic Society/European Respiratory Society criteria for standardization^24^. Best forced expiratory volume in 1 second (FEV_1_) and forced vital capacity (FVC) values were selected for data analysis. Spirometric data were obtained on site by clinical technicians. The percentage of predicted values for FEV_1_ and FVC were calculated from the following equations obtained in a representative Korean population^25^.

$${\rm{Predicted}}\,{\rm{FVC}}\,{\rm{in}}\,{\rm{men}}=-4.8434-(0.00008633\times {{\rm{age}}}^{2}\,[{\rm{year}}])+(0.05292\times {\rm{height}}\,[{\rm{cm}}])+(0.01095\times {\rm{weight}}\,[{\rm{kg}}])$$

$${\rm{Predicted}}\,{\rm{FVC}}\,{\rm{in}}\,{\rm{women}}=-3.0006-(0.0001273\times {{\rm{age}}}^{2}\,[{\rm{year}}])+(0.03951\times {\rm{height}}\,[{\rm{cm}}])+(0.006892\times {\rm{weight}}\,[{\rm{kg}}])$$

$${\rm{Predicted}}\,{{\rm{FVC}}}_{1}\,{\rm{in}}\,{\rm{men}}=-3.4132-(0.0002484\times {{\rm{age}}}^{2}\,[{\rm{year}}])+(0.04578\times {\rm{height}}\,[{\rm{cm}}])$$

$${\rm{Predicted}}\,{{\rm{FVC}}}_{1}\,{\rm{in}}\,{\rm{women}}=-2.4114-(0.0001920\times {{\rm{age}}}^{2}\,[{\rm{year}}])+(0.03558\times {\rm{height}}\,[{\rm{cm}}])$$

### Measurement of insulin resistance

The homeostasis model assessment of insulin resistance (HOMA-IR) value was used as a measure of insulin resistance and was defined as (fasting insulin [μIU/mL] × fasting glucose [mg/dL])/405^26^.

### Statistical analysis

All continuous variables are presented as means and standard deviations. Analysis of variance (ANOVA) was used to compare mean values for continuous variables. All multivariate analyses were performed with linear regression and multiple logistic regression within the SURVEY procedure in SAS software (Statistical Analysis System, Version 9.3, SAS Institute Inc., Cary, NC, USA). Statistical significance was set at P < 0.05.
